# Fatal Influenza A(H1N1)pdm09 Encephalopathy in Immunocompetent Man

**DOI:** 10.3201/eid1906.130062

**Published:** 2013-06

**Authors:** Marie Simon, Romain Hernu, Martin Cour, Jean-Sébastien Casalegno, Bruno Lina, Laurent Argaud

**Affiliations:** Hospices Civils de Lyon, Lyon, France (M. Simon, R. Hernu, M. Cour, J.S. Casalegno, B. Lina, L. Argaud);; Université Claude Bernard Lyon 1, Lyon (M. Cour, B. Lina, L. Argaud);; Institut National de la Santé et de la Recherche Médicale, Lyon (M. Cour, L. Argaud);; Centre National de Référence des Virus Influenza Région Sud France, Lyon (J.S. Casalegno, B. Lina)

**Keywords:** influenza A(H1N1)pdm09, influenza, influenza virus, viruses, encephalitis, encephalopathy, neurologic complication, cerebral venous thrombosis, seizure, adult

## Abstract

We report an immunocompetent patient who had fatal encephalopathy after mild influenza. He rapidly died after unusual symptoms related to intracerebral thrombosis and hemorrhage. A brain biopsy specimen was positive for influenza A(H1N1)pdm09 virus RNA, but a lung biopsy specimen and cerebrospinal spinal fluid samples were negative.

Influenza-related neurologic complications are rare, especially in immunocompetent adults. The clinical signs and severity of this pathology are variable. We report a life-threatening specific complication of influenza A(H1N1)pdm09 infection that was responsible for lethal central venous thrombosis.

## The Study

A previously healthy 26-year-old man from northern Africa was admitted to our emergency department in Lyon, France, in November 2009, during the peak of influenza A(H1N1)pdm09 infection in France (*1*), because of cephalalgia, confusion, and lethargy. A Glasgow Coma Score was 12. He had no history of influenza vaccination. Initial symptoms (fever, cough, and myalgia) began a week before admission. Several members of his family had similar symptoms. There were no risk factors indicative of a complicated disease. Body temperature at admission was 36.8°C, and he had no respiratory distress or signs of shock. Results of a chest radiograph were normal.

During the first hours after admission, the patient lost consciousness (Glasgow coma score 3), which was associated with a seizure. His pupils were anisocoric and nonreactive to light. Intubation was then required to protect the airways. A cranial computed tomographic (CT) scan showed thrombosis of the superior sagittal sinus associated with 3 cerebral hematomas (left frontal and bilateral parieto-occipital) and diffuse cerebral edema with signs of increased intracranial pressure (Figure).

**Figure:**
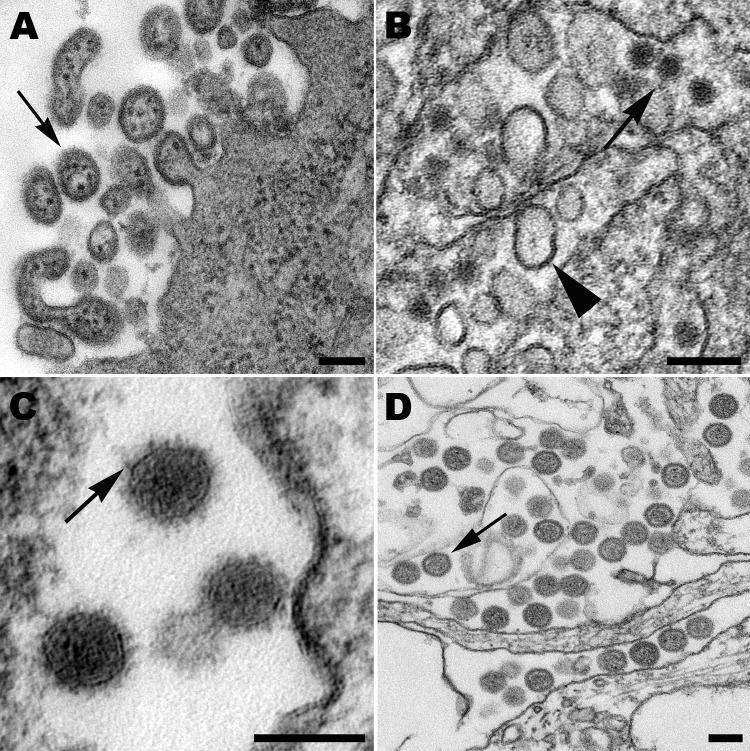
A) Noncontrast cranial computed tomographic (CT) scan of a 26-year-old immunocompetent man with influenza, showing diffuse cerebral edema (Ed) and bilateral parieto-occipital hematoma (H). B) Cranial CT scan with contrast injection, showing diffuse cerebral edema (Ed) and cord sign (arrow) related to a venous thrombosis (VT) of the superior sagittal sinus.

Biologic results showed an increased neutrophil count (14.5 ×10^9^ cells/L), thrombocytopenia (25 × 10^9^ platelets/L), and an inflammatory syndrome (C-reactive protein level 49.7 mg/L). There was no renal dysfunction and no increases in levels of serum lactate or abnormalities in levels of cardiac, hepatic, and pancreatic enzymes. Toxicology screening showed no alcohol or drugs present. Results of thrombophilia screening (standard blood coagulation tests and tests for antibodies against thrombin III and phospholipid) were negative.

Real-time PCR for nasopharyngeal swab specimens rapidly confirmed influenza A(H1N1)pdm09 infection. Test results for cerebrospinal fluid (CSF) (312,000 erythrocytes/mm^3^, 1,000 leukocytes/mm^3^, glucose level 0.84 mmol/L, and protein level 2.7 g/dL) were not informative because of massive hemorrhaging. Results of real-time PCR for CSF were negative for influenza A(H1N1)pdm09 virus, herpes simplex virus (HSV1 and HSV2), and enterovirus. Results of serologic analyses for infectious agents often associated with encephalopathy (cytomegalovirus, Epstein-Barr virus, HSV, rubella virus, enterovirus, and *Mycoplasma pneumoniae*) were negative. The patient was also negative for HIV. Surgery was not considered because the neurologic condition was irreversible. Two electroencephalographic records showed no cerebral activity, confirming this poor prognosis. The patient died 72 hours after admission.

An autopsy was performed. Macroscopic examination showed a congested and edematous brain. Thrombosis of the superior sagittal sinus was caused by a platelet–fibrin thrombus. Acute subarachnoid hemorrhage was found with multiple intraparenchymal infarcts involving the frontal and parietal lobes. Cerebral tonsillar and bilateral uncal herniations were noted. Inflammatory infiltrates were scarce, and few perivascular lymphocytes were found. Immunohistochemical analysis showed no macrophagic infiltration, suggesting recent (<3 days) infarcts. A brain biopsy specimen was positive by real-time PCR for influenza A(H1N1)pdm09 virus RNA, but a lung biopsy specimen was negative by real-time PCR and culture.

## Conclusions

We describe fatal encephalitis in the form of central venous thrombosis associated with influenza A(H1N1)pdm09 virus infection in an immunocompetent man. Influenza-associated encephalopathy (IAE) is a rare complication of a common disease and is more frequently described in children (1–4 cases/100,000 person-years) (*2**,**3*). In children, IAE related to seasonal influenza results in variable relapse and a mortality rate as high as 30% (*3*). In adults, seasonal IAE is infrequent and poorly characterized (*4*). Symptoms in the patient were typical of neurologic disorders of IAE, including disorientation, meningismus, agitation, seizures, and coma (*2**,**4*). The incidence of neurologic complications from influenza A(H1N1)pdm09 has not been determined, and it is not clear whether this pandemic was associated with increased neurologic complications compared with those of seasonal influenza (*5*).

Several series of neurologic complications, especially those involving children, have been published and occasionally reported poor prognosis (*5**–**7*). To our knowledge, only a few reports have described cases in adults (*8**–**12*). A 20-year-old man had refractory seizures in association with malignant edema and survived with severe neurologic sequelae (*8*). A 22 year-old woman showed development of persistent Parkinsonian features and hypothalamic dysfunction manifestations after IAE (*9*). As in our patient, these 2 patients had no respiratory distress. A 40-year-old patient had prolonged hypoxemia secondary to the acute respiratory distress syndrome (ARDS) associated with acute hemorrhagic leukoencephalitis, which was responsible for severe disability (*10*). Two patients had fatal cerebral edema and transtentorial brain herniation syndrome associated with ARDS and renal failure (*12*).

The pathogenesis of IAE remains unclear (*2*). As demonstrated in the case reported, influenza virus is rarely detected in CSF and pleiocytosis is often absent, suggesting that direct invasion by influenza A virus is unlikely to be the cause of encephalopathy (*2*). Hematogenous spreading is unlikely because viremia is rare in humans, and influenza virus–associated neurotropism has not been demonstrated. In addition, influenza virus viremias are often associated with ARDS caused by massive virus replication in the lungs during infection, but our patient had no pulmonary infection.

Pathogenesis might be related to a hyperactivated cytokine response in the context of a systemic inflammatory response syndrome. In patients with influenza encephalopathy, levels of proinflammatory cytokines and soluble cytokines receptors are increased in serum and CSF (*13*). Symptoms may be caused by cytokines, which could cause direct neurotoxic effects, cerebral metabolic changes, or breakdown of the blood–brain barrier (endothelial injury) (*14*). However, lack of benefit from use of steroids or intravenous immunoglobulin for influenza-associated encephalopathy does not support this potential mechanism (*4*).

Neuroimaging findings by CT or magnetic resonance imaging (MRI) for this pathogenic process usually include focal or diffuse cerebral edema, necrosis (especially in children), demyelinization or hemorrhagic injury (*2*). Patients exhibiting neuroradiographic abnormalities have more severe sequelae or higher mortality rates than patients with normal CT or MRI results (*4*). Influenza A(H1N1)pdm09 virus infections might be associated with increased abnormalities detected by MRI compared with those associated with seasonal influenza (*6*). To our knowledge, there has been no report of IAE related to cerebral venous thrombosis, including influenza A(H1N1)pdm09 infections.

Histologic abnormalities of the brain are often absent in patients who die with clinical signs of IAE (*15*). Influenza virus antigens are generally not detected in the brain (*8*). We found evidence of direct viral neuroinvasion and positive results by real-time PCR for a brain biopsy specimen for influenza A(H1N1)pdm 09 virus RNA, which indicates microbiologically documented encephalitis associated with influenza A(H1N1)pdm09 infection.

In summary, IAE is a rare complication of a common disease that was also diagnosed during the influenza A(H1N1) 2009 virus pandemic. Cases in adults usually remain mild, but our results show that clinicians should be alert to potential neurologic complications of influenza, even without respiratory symptoms. The severity of neurologic sequelae warrants investigation of these sporadic cases. Increased knowledge of host–virus interaction in the brain and necropsy studies of cases with cerebral involvement could provide better understanding of this interaction.
